# Bioimpedance-based Authentication of Defrosted Versus Fresh Pork at the End of Refrigerated Shelf Life

**DOI:** 10.2478/joeb-2022-0017

**Published:** 2023-01-14

**Authors:** Daniel E. Osen, Sisay Mebre Abie, Ørjan G. Martinsen, Bjørg Egelandsdal, Daniel Münch

**Affiliations:** 1 Department of Physics, University of Oslo, 0316 Oslo, Norway; 2 Department of Clinical and Biomedical Engineering, Oslo University Hospital, 0372 Oslo, Norway; 3 Faculty of Chemistry, Biotechnology and Food Science, Norwegian University of Life Sciences, 1432 Ås, Oslo, Norway; 4 Animalia, Norwegian Meat and Poultry Research Center, 0513 Oslo, Norway; 5 Faculty of Ecology and Natural Resource Management, Norwegian University of Life Sciences, 1432 Ås, Oslo, Norway

**Keywords:** Bioimpedance, freezing, thawing, meat, phase angle, P_y_-parameter, food labelling

## Abstract

Correct food labeling is a legal requirement and helps consumers to make informed purchasing choices. Mislabeling defrosted meat as fresh is illegal in the EU. However, there are no standardized technologies to authenticate fresh versus defrosted meat. We address this by testing if bioimpedance-based measurements can separate defrosted meat from refrigerated-only meat at the end of shelf life, i.e., when also fresh meat shows deterioration. Pork sirloin samples from 20 pigs were first tested at 12 days postmortem (‘fresh group’). This time point was chosen to represent a typical use-by date for refrigerated storage of fresh pork. Then, all samples were transferred to a -24°C freezer for 3 days and thawed for 2 days before final testing (‘frozen-thawed group’).

Bioimpedance analyses (BIA) were done in a frequency range of [10^2^-10^6^ Hz]. Weight, pH and electrode positioning were assessed to test for potential confounding effects. Statistics for treatment dependent differences were based on the established P_y_ parameter and phase angle, which were extracted from the BI spectra. We found that using bioimpedance testing with tetrapolar electrodes, P_y_ and phase angle allowed almost complete separation of fresh and previously frozen samples. However, within the whole sample population, there was some overlap between the spectra of fresh and frozen samples. Yet, based on P_y_, only one fresh sample (5% of N_total_=20) fell in the lowest P_y_ class with all the frozen samples. We used a multifactorial design that allowed to test the effects of potential confounding factors, such as electrode positioning and meat quality parameters. We found a relatively low explained variance for the P_y_ parameter, indicating that confounding effects from other factors or quality defects in fresh pork may affect the detection capacity of bioimpedance-based authentication of fresh pork. Our data, therefore, suggest that reliable fresh-label authentication with bioimpedance testing should be based on testing a small number of samples to represent a specific lot of pork that is to be inspected.

## Introduction

Labeling requirements by food authorities provide important tools to ensure food safety standards and enable consumers to make informed choices about the food they buy. In the European Union (EU) and countries adopting EU laws, current food labeling requirements are established by regulation (EU) No 1169/2011 [1]. Concerning fresh and defrosted meat products, the regulation specifies that the freezing and later defrosting of certain foods, especially meat and fishery products, limits their possible further use and may also have an effect on their safety, taste and physical quality. Hence, producers are required to include a “defrosted” label, if meat has been defrosted as opposed to “fresh” meat, which only has been refrigerated. Yet, fraudulent labeling is difficult to reveal as no test standard is established for fresh versus defrosted authentication. Moreover, existing laboratory methods, e.g., based on detection of marker proteins that are released during a freeze-thaw cycle, provide limited reliability for relevant freezing conditions (see e.g., [2] for a summary). To help establishing a sufficiently reliable alternative sensor technology we investigate if bioimpedance can detect differences between fresh and defrosted pork meat under conditions that resemble real-life settings, e.g., by comparing defrosted pork with pork after extended refrigeration periods, as encountered, e.g., in supermarkets. We hypothesized, that bioimpedance testing allows to separate fresh from defrosted meat, despite the effects of other factors, e.g., a progressive tissue deterioration during long refrigeration storage.

Freezing is one of the most common and widely used preservation methods. However, ice crystal formation during freezing destroys cell membranes and other cellular structures [3, 4]. Such deterioration can be alleviated – but not entirely prevented – by faster freezing, which is typically linked to the formation of smaller ice crystals (e.g., [5]). However, inevitable ice crystal formation also causes secondary, deleterious effects, e.g., by increasing local salt concentrations, denaturing of proteins, and the release of intracellular proteins, fats, and minerals [6]. Importantly, freeze-thawing also reduces the water holding capacity and hence “juiciness” of meat. More specifically, higher drip loss in defrosted meat can be due to cellular breakdown, changes in the hydrophilic/hydrophobic balance of filamentous protein complexes in the muscle cells, and reduced retention of extracellular moisture during thawing [7]. The exudate that is lost during thawing is typically referred to as thaw loss. Thaw loss is typically assessed by weighing the fluid that is released, e.g., when meat is held in a bag during an entire freeze-thaw cycle. While even a single freeze-thaw cycle will inevitably cause detectable quality decline, also long-term refrigeration is linked to some deterioration, which is why food authorities require a “use-by” label.

Electrical impedance measurements are sensitive to structure and composition changes of biological tissues. Several studies have shown that measuring electrical bioimpedance, i.e., the passive electrical properties of meat, allows detecting differences between fresh and frozen-thawed meaty products from fish [8], chicken [9], and pork [5,10]. However, information is scarce on potentially confounding effects, on the selection of suitable bioimpedance parameters and test-setups, and on the applicability for different types of meat products and storage history. Moreover, it is not clear, whether differentiation between fresh and frozen-thawed meat products can be done reliably throughout product shelf-life, as tissue damage also progresses through refrigerated storage.

Electrical impedance is an inexpensive, potentially noninvasive, and relatively simple method to analyze electrical properties of materials by inducing alternating electrical signals at different frequencies into them, and by recording the response signals. Measuring impedance in the range from 100 Hz to 1 MHz covers two different dispersion regions known as the α and β dispersion. The α dispersion is influenced by counter ion effects near the membrane surface, ion diffusion and dielectric losses [11]. The β-dispersion is mostly influenced by the polarization of membrane structures. Thus, measuring the passive electrical behavior of meat in these frequency ranges provides information about electrolyte distribution (“meat juices”) and about the capacity to accumulate charges at intact cellular structures [12, 13, 14]. Hence, the suitability of bioimpedance testing for fresh versus frozen-thawed detection may be attributed to the changes in structure and integrity of animal cells upon freeze-thawing.

The measured impedance response is often characterized by four Cole parameters that are extracted by circular regression: the resistance at high and low frequency, R_∞_ and R_0_ respectively; ω is the angular frequency, the characteristic frequency $f_{C}=(2\cdot \pi \cdot \tau {)}^{-1}$and the distribution and interaction parameter, α [15].


$$Z=R_{\infty }+\frac{R_{0}-R_{\infty }}{1+(j\omega \tau {)}^{\propto }}$$

This mathematical expression has been widely utilized to represent the frequency-dependent electrical impedance of biological tissues. It describes a complex nonlinear function of frequency that can be represented in the impedance plane, which is perpendicular to the frequency plane (see [16] for more details about these concepts).

The normalized extent of the β-dispersion is termed as the P_y_ value [13] and has been used previously to assess different meat quality features.


$$P_{y}=\frac{R_{0}-R_{\infty }}{R_{0}}$$

An alternative analyses approach that has not been used previously might be based on extracting phase angle data. Briefly, whenever an electric current is applied to biological tissues, the capacitive nature of the cell membrane delays the buildup of an electric potential across the membrane. This characteristic creates a phase shift between current and voltage. Thus, the phase angle measured during bioimpedance measurement yields the relation between resistance (R) and reactance (X_C_) of the sample. The phase angle ranges between 0° and 90°, and can inform about tissue integrity or quality, sample dimension, and also the movement and distribution of water between intra and extracellular spaces. A phase angle of 0° indicates a circuit with only resistive characteristics, i.e., in a system with no or completely degraded cell membranes. 90°, in contrast, indicates a circuit with only capacitive characteristics, i.e., with cells having no extracellular fluid resistance and only membrane capacitance [17,18]. Therefore, in the medical sector, phase angle is considered a possible global marker of health that can be used for evaluation of cell membrane function. Yet, the exact biological meaning of the parameter is still not fully understood [19].

Here we aim to investigate how bioimpedance based separation of defrosted versus fresh-chill stored products may be impaired by long refrigerated storage, i.e., when also the fresh product will experience some deterioration. Furthermore, we test the robustness of bioimpedance parameters against other potential confounding factors, such as the positioning and orientation of the electrodes, key meat quality parameters such as pH and drip loss. Lastly, we explore the use of different impedance related parameters for frozen-thawed discrimination of pork sirloin samples.

## Materials and methods

Fresh pork sirloin (Longissimus thoracis et lumborum) cuts (N=22) were obtained at day 4 postmortem from a research meat cutting plant (Oslo, Norway). Cuts were individually packed in vacuum plastic bags and stored for 8 days in a fanned cold storage room at a temperature of around 3 °C.

At day 12 postmortem, in random sequence, each meat sample was quicky transferred to another room (ca. 20 °C) for measurements of potential confounding factors. First, the weight of the cuts was determined and ranged between 269 g to 398 g. To assess drip loss, we also weighed the exudate (drip), that had accumulated in the bag. The pH was measured using a Knick Portamess pH meter. The pH of the drip loss was used rather than the pH of the meat as the measurement stabilizes quicker in solutions. Samples were put into new plastic bags and were transferred again to the cold storage room. Thermocouples were inserted into two of the samples to monitor temperature.

After the sirloin cuts were acclimatized again to the temperature of the fanned cold storage room, bioimpedance was measured using a tetrapolar probe and a Zürich Instruments MFLI (Zurich Instruments AG, Switzerland), with an applied voltage of 400 mV rms and 100 frequency points from 100 Hz to 1 MHz (“fresh group”).

Once bioimpedance measurements had been completed, the samples were transferred to a fanned freezer room (set to -24 °C) for three days. Finally, samples were moved back into the cold storage room for thawing and temperature equilibration. After two days, a full freeze-thaw cycle was confirmed through reading the temperature loggers and bioimpedance measurement were taken (“frozen-thawed group”). The bioimpedance measurements of the frozen-thawed samples were done in the same sequence as done for the fresh samples.

To calculate the P_y_ parameter for each impedance spectrum, a least square curve fitting of the Cole equation was performed (Cole, 1940). Specifically, the equation was fitted to the experimental data using iterative methods in the frequency or impedance domains. Thus, the extraction of the Cole parameters was performed using both, the non-linear circular fitting [39, 40, 41] and impedance modulus spectrum curve fitting. In cases where samples clearly lacked a frequency dependent dispersion, Cole fitting was not attempted. Instead, P_y_ was estimated using the impedance modules at f = 1000 Hz and f = 1 MHz, which replaced R_0_ and R_∞_, respectively.

## Ethical approval

The research related to animals use has been complied with all the relevant national regulations and institutional policies for the care and use of animals.

## Data analysis

Impedance, mass, drip loss, and pH measurements were conducted, and the results are reported graphically and as mean value ± standard deviation. The analysis of variance (ANOVA) and Mann Whitney Wilcoxon Test were conducted using Minitab and MatLab statistical software with significance at the 5% level (p < 0.05).

## Results

**Figure 2:**
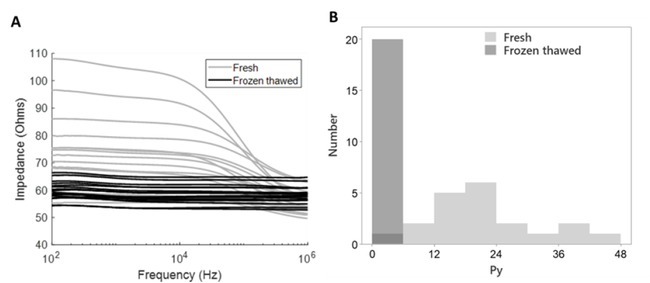
Individual impedance response spectra and P_y_ values indicate almost complete separation of refrigerated and subsequently frozen-thawed pork samples (N=20 for each test group and one specific electrode position). A. Impedance spectra before freezing (refrigerated) and after thawing (frozen-thawed). B. Histogram plot of P_y_ values for all pork sirloin samples, as extracted from one specific position measurement.

Electrical impedance testing was done twice for each sirloin sample: first around expiry date and then, after a single freeze-thaw cycle. Fig. 2A shows the individual impedance spectra for all 20 samples, measured as refrigerated only and when frozen-thawed. Generally, the impedance modulus in the sub 100 kHz range was higher for refrigerated samples and reduced after samples were exposed to freeze-thawing. However, assessing the raw impedance spectra revealed a slight overlap between the two test groups. We then explored if separation between refrigerated and frozen-thawed samples was frequency dependent, and contrasted impedance modulus responses for each tested frequency (see Material and Methods) using the full frequency sweep spectrum (Mann Whitney Wilcoxon Test, data not shown). The statistical comparison of the treatment groups revealed various significance levels for different parts of the frequency range with the highest levels of significance (p < 0.001) typically obtained at lower frequencies between 1 kHz and 500 kHz, and no significant treatment effects (p > 0.05) at frequencies above 500 kHz.

To further explore freezing-dependent separation of bioimpedance responses, we calculated the P_y_ parameter, an established proxy for the β-dispersion, i.e., the typical frequency dependent impedance decline that is observed above ca. 10 kHz. The histogram plot for individual P_y_ values (Fig. 2B) shows a large P_y_ variation within the refrigerated group, while the distribution of frozen-thawed samples is more homogenous, and entirely restricted to the smallest P_y_ class, ([0, 1, 2, 3, 4, 5, 6]). Compared to raw impedance spectra (Fig. 2A), P_y_-based analyses reveals a more complete treatment-dependent separation, with only one refrigerated sample being observed in the smallest P_y_ class.

We next tested for factors that might compromise impedance-based authentication of fresh samples by analyzing P_y_ response data with a full factorial design (Table 1). To this end, we included treatment (refrigerated vs. frozen-thawed), and two electrode positioning variables as main factors, and individual sample weight, drip loss, and pH as co-variates. Our analysis confirms a highly significant effect of treatment (refrigerated/frozen-thawed), and also a significant effect of sample weight. No significant effects were found for electrode position, drip loss and pH, nor did we detect significant interaction among main factors and covariates. Yet, while the main factor “freezing treatment”, showed the highest explained variance among test variables, a large part of P_y_ variance is not explained by the tested parameters (compare Fig. 2B for the large P_y_ variation in the refrigerated group). However, we report that selection of covariates can greatly affect explained variance by freezing treatment. For example, if drip-loss is removed from the model the explained variance increased to 0.505 for the main factor (refrigerated/frozen-thawed, data not shown).

**Table 1:** Summary of the analyzed full factorial design (ANOVA) with P_y_ as response and a two-level factor encoding for potentially confounding effects (R/FT = refrigerated/frozen-thawed; On/Al = electrode position ‘on-top’ into back muscle/see Fig. 1C, D versus ‘along’, through back muscle/see Fig. 1A, B; Pos = Position of electrode, left and right side of the sample; * = p > 0.2).

P_y_	Variables	Explained variance (adj.) (%)	p-value
Main factors	R/FT	12.8	<0.001
	On/Al	-	*
	Pos	0.2	0.051
Interaction	F/FT. On/Al	-	*
	F/FT. Pos	1.4	0.106
	On/Al.Pos	-	*
Covariates	Driploss (22.5 ± 1.2g)	-	**
	Weight (335.4 ±7.4g)	3.7	0.009
	pH (5.22 ± 0.01)	-	*
Error		79.5	-

**Figure 1:**
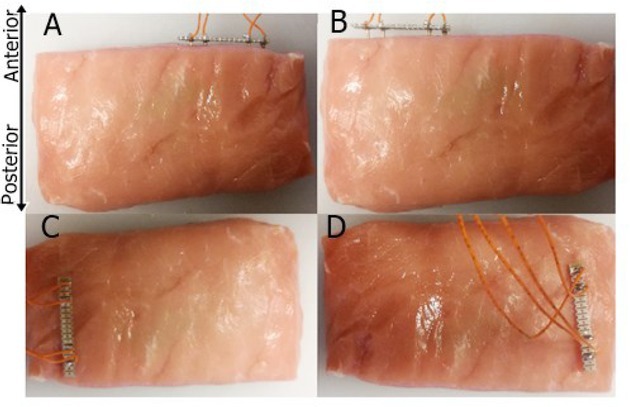
Measuring the bioimpedance of sirloin cuts. Four different positions were chosen for the electrode, two with the electrode inserted perpendicular to the anterior-posterior axis of the LTL (A, B; approximately also perpendicular to the main muscle fiber direction) and two positions along the anterior-posterior axis (C, D; approximately in line with the main muscle fiber direction C, D). The side facing the camera is the dorsal side of the LTL muscle (depicted axes: anterior/posterior & left/right).

**Figure 3:**
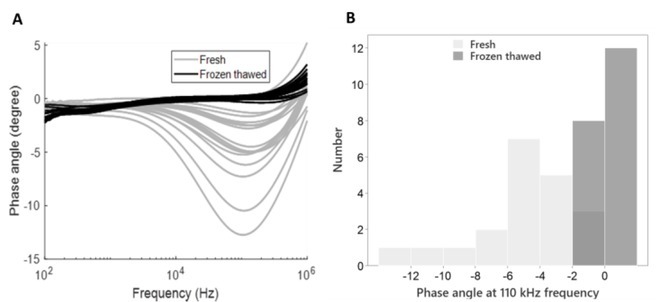
Individual impedance phase angle responses in pork before and after freezing-thawing (N=20 per treatment group). A. The phase angle of refrigerated and frozen-thawed meat measured in one specific position. B. The distribution of phase angle values at 110 kHz reveals apparent differences between refrigerated and frozen-thawed samples.

Lastly, plotting the phase angle spectra revealed another potential difference between refrigerated and frozen-thawed samples (Fig. 3A). Like our analyzes of the frequency dependent impedance responses (compare Fig. 1A), we explored frequency dependent treatment separation by contrasting phase angle responses for each tested frequency. We found highly significant differences (Mann Whitney Wilcoxon Test, p < 0.001) for current frequencies between 10 kHz and 500 kHz, while treatment effects became undetectable (p > 0.05) in frequency bands below 10 kHz and above 500 kHz. Figure 3B shows the distribution for a single frequency (110 kHz), for which the spectrum (Fig. 3A) indicates maximum separation between treatment groups.

The phase angle histogram for 110 kHz reveals only a minor overlap for one class ([0;-2]). Again, compared to the wider distribution within the refrigerated group, the distribution of frozen-thawed samples was compressed and limited to the smallest phase angle classes (compare Fig. 2B). Phase angle difference among the groups was found to be common to all four position measurements, suggesting that phase angle-based treatment separation is robust against changed electrode positioning.

## Discussion

To the best of our knowledge, bioimpedance has not been used before to test if refrigerated pork around expiry date can be still separated from frozen-thawed pork. Here we establish that bioimpedance-based testing reveals significant differences between groups of refrigerated versus frozen-thawed pork. Significant treatment dependent differences were detected for three selected impedance parameters, impedance modulus, P_y_ and phase angle. Confounding effects that might impair “fresh” pork authentication were detected only for product weight, and not for, e.g., drip loss, pH, and electrode positioning.

Our data corroborates previous bioelectric studies, which have contrasted groups of refrigerated-only with frozen-thawed samples, and proposed bioimpedance-based testing as a suitable method for authentication of fresh meaty foods or fish [20, 21, 22, 23]. Typically, such bioelectric studies were based on testing for resistance and reactance individually, on the impedance modulus at distinct frequencies, or on parameters that reflect the frequency modulation of the impedance response, such as P_y_ [10,13,23,24]. The significantly reduced impedance response, expressed as P_y_, that we found after freezing (compare Fig. 2 and Table 1) is typically believed to result from an overall destruction of cell membranes and other capacitive elements, as well as from increased amounts of free electrolytes in extracellular spaces [9]. While data that directly links micro-structural damage with changed bioimpedance response after freezing is scarce, we previously demonstrated the vast extend of ice crystal formation in frozen pork with cryo-scanning electron microscopy and a greatly reduced bioimpedance response measured in similarly freeze-treated samples [10].

Apart from previously described impedance parameters, we also identified a marked effect of freeze treatment on phase angle (Fig. 3A, B). Treatment related separation was most prominent in the higher frequency range, which corresponds to the β-dispersion range [13, 24]. Based on the relation of phase angle with reactance and resistance, lower phase angle appears to be consistent with low reactance, typically linked to the breakdown of cellular compartments, including membranes [25]. While phase angle parameters, apparently have not been previously described for detection of meat quality decline caused by freezing, a recent report describes a relevant nutritional assessment and evaluated the risk of various diseases, such as locomotive syndrome (LS), liver cirrhosis etc. [26, 27, 28, 29]. In sum, in our feasibility study with a limited sample number we did not attempt to benchmark the capacity for fresh versus frozen detection for the three different parameter types we analyzed in our study (compare Figs. 2, 3). However, based on our findings we propose to include also phase angle – or more complex parameters that not only describe the amplitude at a given frequency – with future, larger-scale studies. These can allow assessing how performance for authentication of fresh meat depends on impedance parameter selection.

While our analyses reveal highly significant treatment effects, we found a few fresh samples with impedance modulus, P_y_, and phase angle values relatively close to the frozen-thawed samples. There are several potential explanations for reduced impedance responses also in “fresh”’, refrigerated-only samples. Firstly, changes in the meat’s protein matrix, including the degradation of cellular membrane proteins, are inescapably linked to normal postmortem events that mark the transition from living muscle tissue to meat, as well as later events that accompany meat aging [30,31]. One of the hallmark features of early postmortem changes is the formation of drip-channels, which are formed, when intracellular fluids leave muscle cells [31,32]. Such drip loss is thought to result from changes to the water holding capacity of myofilaments and cellular membrane leakage [33]. Secondly, apart from normal post-mortem quality changes, tissue degradation can be aggravated by common pork meat defects, including the so-called PSE-like or “destructured pork” and known heritable defects (PSE/hal+ pork, acid/RN–pork, [34,35]). Afflicted meat can exhibit vastly diverse degrees of cellular and fiber disintegration, often causing excessive drip loss. Relatively low pH values, sometimes below pH 5.4, may indicate the presence of such quality anomalies among the samples we have tested. Consequently, common quality defects may explain for the wide P_y_ and phase angle distributions we observed (compare Fig. 2B, 3B), and also for the few samples that – even before freezing – exhibited impedance responses close to what we typically detected first after one freeze-thaw cycle (compare Fig. 2).

Based on the present findings, we can only speculate on how fresh pork quality defects may impair bioimpedance based authentication of fresh pork. However, with a recent study and a larger sample set (N>80), we could already establish links also between reduced bioimpedance response and indicators of quality defects in fresh pork [36]. Combining findings from the latter with this study may suggest that P_y_ variation for pork in fresh vs. defrosted tests may roughly comprise two entities, the almost distinct populations of fresh versus defrosted samples and a large, more continuous P_y_ distribution within the refrigerated group. Accordingly, known mechanisms of cellular degradation in fresh pork [36, and references therein] may interact with cellular deterioration during freezing. The pronounced, likely quality dependent, P_y_ variation in fresh pork may therefore account for the relatively low explained variance we found for treatment (compare R/FT in Table 1), despite the treatment group separation we show in Fig. 2.

## Methodological considerations

Assessing the impact of potentially confounding factors will be critical for establishing bioimpedance testing for ‘fresh-label’ authentication. Here, testing for electrode positioning can be relevant, as robustness against “handling errors” will be a prerequisite for the use of bioimpedance testing also by non-experts, in particular persons without prior knowledge of muscle anatomy.

Importantly, we did not detect an effect of electrode positioning, when electrodes were placed either along the longitudinal axis of the LTL muscle or perpendicular to it (compare Fig. 1 and ‘On/Al’ in Table 1). Such positioning roughly reflects measuring along and across main fiber direction, for which a previous study reported effects [37, 38]. However, our data suggests that such effect might be negligible compared to the large effect of freezing treatment. Yet, we also found a non-significant trend, when placing the electrode either left or right from a sample’s mid-point (both perpendicular to the longitudinal LTL axis, see Fig. 1 and ‘Pos’ in Table 1). Alternatively, such effect can be rather conferred by dimension, i.e., non-random thickness-variation within samples. Similar, the confounding effect of weight we detected (Table 1), might suggest, that controlling for confounding effects of sample dimension may improve bioimpedance-based detection of freeze-treatment effects.

The comparably smaller effects of electrode positioning and weight, together with a failure to detect influences from sample pH and drip loss support that impedance-based detection of fresh vs. frozen differences is rather robust against potential bias from the other tested parameters. Yet, controlling for potential bias might improve impedance-based detection of freeze-treatments, potentially resulting in a more complete separation of refrigerated and frozen-thawed samples.

## Conclusion

Freezing, but also post-slaughter refrigerated storage lead to cellular deterioration, which cause changes in the muscle’s impedance. Impedance spectroscopy and a tetrapolar electrode assembly can be suitable for fresh vs. frozen-thawed detection in pork meat. Such testing can be based on P_y_ and phase angle that both show almost complete separation for fresh and frozen-thawed samples, even at the end of the product’s shelf life. Therefore, bioelectrical impedance has a potential to be used as a rapid quality control method for fresh and frozen pork.
